# Extraintestinal Seeding of *Salmonella enterica* Serotype Typhi, Pakistan

**DOI:** 10.3201/eid2703.200464

**Published:** 2021-03

**Authors:** Seema Irfan, Mohammad Zeeshan, Salima Rattani, Joveria Farooqi, Sadia Shakoor, Rumina Hasan, Afia Zafar

**Affiliations:** Aga Khan University. Karachi, Pakistan

**Keywords:** *Salmonella enterica* serovar Typhi, *Salmonella* Typhi, bacteria, extensively drug-resistant, XDR, antimicrobial resistance, extraintestinal seeding, complications, enteric fever, enteric infections, Pakistan

## Abstract

We evaluated *Salmonella enterica* serotype Typhi strains isolated from all body sites in Pakistan during 2013–2018. Despite an increase in overall number of localized, extensively drug-resistant *Salmonella* Typhi in organ infections during 2018, there was no increase in the proportion of such isolates in comparison with non–extensively drug-resistant isolates.

*Salmonella enterica* serotype Typhi is a major pathogen affecting populations from low- and middle-income countries that generally lack clean, potable water and good sanitary disposal systems (*1*). The global incidence of enteric fever is ≈21 million cases annually, and there are ≈200,000 typhoid-related deaths/year (*2*). Pakistan is among the high-burden countries that has reported annual incidence of 493.5 case/100,000 persons (*3**–**5*). Seeding of deep-seated organs by *Salmonella* Typhi, resulting in bone and soft tissue infections and splenic and hepatic abscesses, has been reported (*6**–**9*). Extensively drug-resistant (XDR) *Salmonella* Typhi, a strain resistant to 5 groups of antimicrobial drugs, including third-generation cephalosporins (*10**,**11*), has emerged in 2 cities in the southern part of Sindh Province and further disseminated to other parts of Pakistan, raising concern for persistence of the organism in hosts because of delays in appropriate therapy.

During 2018, a sudden increase in isolation frequency of XDR *Salmonella* Typhi from clinical samples other than blood, stool, and urine in Pakistan was observed. We conducted a study to determine if there was a true increase in the proportion of extraintestinal XDR *Salmonella* Typhi infections compared with non-XDR infections.

## The Study

This study was conducted at the clinical microbiology laboratory at Aga Khan Hospital (Karachi, Pakistan). After approval was obtained from the Ethical Review Committee at Aga Khan Hospital, all reports of clinical specimens that showed growth of *Salmonella* Typhi during January 2013–December 2018 were extracted from the laboratory database and included in the study. The frequency of isolation of the organism from extraintestinal organ infections was compared with that of blood/bone marrow, stool, and urine. In addition, informed consent and detailed history were obtained by telephone from patients who had XDR *Salmonella* Typhi isolated from sites other than blood/bone marrow, stool, and urine during 2018.

*Salmonella* Typhi were identified by using conventional biochemical reactions and API 20E (bioMérieux, https://www.biomerieux.com) and then confirmed by serotyping with *Salmonella* antisera (Becton Dickinson, https://www.bd.com). Susceptibility testing was performed by using the disk diffusion Kirby-Bauer method and recent Clinical and Laboratory Standards Institute (https://clsi.org) performance standards. For XDR *Salmonella* Typhi strains, susceptibility was confirmed by using the Vitek2 System (bioMérieux), except for azithromycin, which was reported by using the disk diffusion method. The Pearson χ^2^ test was applied to calculate the statistical significance by using Stata SE 12.1 software (https://www.stata.com).

During the 6-year study period, 8,736 isolates of *Salmonella* Typhi were reported from blood, bone marrow, stool, and urine, and 62 isolates were reported from other body sites (Table 1). Yearly isolation of *Salmonella* Typhi from different body sites gradually decreased during 2013–2017, but during 2018, there was a slight increase. In addition, although XDR *Salmonella* Typhi isolation from blood, feces, and urine had been consistently increasing since the beginning of outbreak, its isolation from other body sites was not observed until 2017. During 2018, these strains emerged from other sterile body tissues and fluids. However, their isolation proportion was not significant compared with non-XDR isolates (Table 1).

**Table 1:** *Salmonella enterica* serotype Typhi isolates from blood, feces, and urine versus other body sites, Pakistan 2013−2018

Characteristic		No. (%, 95% CI) in blood, feces, or urine	No. (%, 95% CI) in other body sites	Total cases	p value
No. ceftriaxone sensitive		5,858 (99.1, 98.9–99.4)	51 (0.86, 0.63–1.10)	5,909	0.011
No. ceftriaxone resistant		2,878 (99.6, 99.4–99.8)	11 (0.38, 0.16–0.61)	2,889	
Total		8,736 (99.3, 99.1–99.5)	62 (0.70, 0.53–0.88)	8,798	
Year	Ceftriaoxne susceptibility				
2013	Sensitive	662 (98.5, 97.6–99.4)	10 (1.49, 0.57–2.41)	672	0.902
	Resistant	0	0	0	
2014	Sensitive	701 (98.7, 97.9–99.6)	9 (1.27, 0.45–2.10)	710	0.873
	Resistant	0	0	0	
2015	Sensitive	881(98.9, 98.3–99.6)	9 (1.01, 0.36–1.68)	890	0.839
	Resistant	0	0	0	
2016	Sensitive	1,167 (99.5, 99.2–99.9)	5 (0.42, 0.05–0.80)	1,172	0.764
	Resistant	21 (100)*	0	21	
2017	Sensitive	1,299 (99.3, 98.9–99.8)	9 (0.69) 24–1.14)	1,308	0.057
	Resistant	526 (100)*	0	526	
2018†	Sensitive	1,155 (99.2, 98.7–99.7)	9 (0.77, 0.27–1.28)	1,164	0.264
	Resistant	2,324 (99.5, 99.3–99.8)	11 (0.47, 0.19–0.75)	2,335	

*Because 100% of extensively drug-resistant *Salmonella* Typhi were obtained from blood, stool, or urine, there was no 95% CI. †A total of 20 (0.57%) of 3,499 *Salmonella* Typhi were isolated from other body sites during 2018.

Most (7/11, 63.6%) cases showing growth of XDR *Salmonella* Typhi from other body sites were in children, but 4 (36.4%) were in adults. The average duration from fever onset until care was sought was ≈2 weeks. For 9 (81.8%) of 11 case-patients, complications came in the form of deep abscesses at body sites such as gluteal muscles, deltoid muscles, spleen, subdiaphragmatic recess, pleural cavity, breast, and fractured site of a limb. One case-patient who had no concurrent conditions had acute meningitis, and another case-patient had acute abdominal pain secondary to intestinal perforation and leading to pneumoperitoneum.

Antimicrobial drugs such as clindamycin, ciprofloxacin, amoxicillin/clavulanic acid, ceftriaxone, and cefepime showed inappropriate coverage and treatment duration against XDR *Salmonella* Typhi and were used for 8/11 (72.7%) patients during the course of illness. One patient was lost to follow up, but 10/11 (90.9%) patients showed marked improvement after appropriate antimicrobial drug treatment (intramuscular meropenem or oral azithromycin) and survived (Table 2).

**Table 2:** Clinical characteristics of 11 patients who had XDR *Salmonella enterica* serotype Typhi isolated from blood, feces, and urine versus other body sites, Pakistan 2013−2018*

Patient ID	Age, y	Diagnosis	Manifestation	Concurrent condition/history	Procedure/specimen	Organism/drug susceptibility pattern	Drug used before culture report	Drug used after culture report/duration, wk	Patient status at follow up
**1**	13	Right gluteal abscess	Right gluteal swelling, high grade fever for 1 mo	None	Incision and drainage/pus aspirate	*Salmonella* Typhi//XDR strain	Oral and IM antipyretics, cefepime, clindamycin, ciprofloxacin	Azithromycin, meropenem/2	Improved
2	17	Right gluteal abscess	Right gluteal swelling, nonresolving fever 2 wk	None	Incision and drainage/pus aspirate	*Salmonella* Typhi/XDR strain	IM antipyretic, amoxicillin/clavulanate	Azithromycin, meropenem/2	Improved
3	55	Splenic abscess	Pain in left upper abdomen, fever for 2 wk	Road traffic accident and suspected splenic injury 3 years ago	Ultrasound-guided aspiration/pus aspirate	*Salmonella* Typhi/XDR strain	Ceftriaxone, metronidazole, antipyretics	Meropenem/not known	Improved
4	10	Right leg abscess	Right leg swelling at fracture site, fever for 2 wk	Right leg fracture	Incision and drainage/pus aspirate	*Salmonella* Typhi/XDR strain	Azithromycin, antipyretics	Meropenem, azithromycin/not known	Improved
5	15	Acute meningitis	Altered level of consciousness, seizures, fever for 2 wk	None	Lumbar puncture/CSF	*Salmonella* Typhi/XDR strain	Ceftriaxone, antipyretics	Meropenem/2	Improved
6	15	Splenic abscess	Pain in left upper quadrant of abdomen, intermittent fever for 1 y	Recurrent episodes of pallor and jaundice for 2 y, positive result for antinuclear antibody	Ultrasound-guided drainage/pus aspirate	*Salmonella* Typhi/XDR strain	Meropenem, antipyretics	Meropenem/3	Improved
7	55	Left pleural effusion	Pain in left side of chest and shortness of breath	Smoked for 30 y, high-grade fever for 3 mo, back pain	Pleural tap, chest tube/pleural fluid	*Salmonella* Typhi/XDR strain	Multiple antimicrobial drugs (unknown), antipyretics	Meropenem/not known	Improved
8	40	Right deltoid abscess	Fever, vomiting, abdominal pain, right arm swelling for 2 wk	None	Ultrasound-guided drainage/pus aspirate	*Salmonella* Typhi/XDR strain, MRSA, *Salmonella* Typhi	Azithromycin, intramuscular antipyretics	Meropenem, azithromycin/2	Improved
9	9	Subdiaphragmatic abscess	Fever, pain in abdomen	NA	NA/pus aspirate	*Salmonella* Typhi/XDR strain	NA	NA	NA
10	16	Ileal perforation and pneumoperitoneum	High-grade fever for 1 mo, abdominal pain and distension	None	Laparotomy, ileostomy/abdominal fluid	*Salmonella* Typhi/XDR strain, *Escherichia coli, Enterococcus* spp.	Multiple empiric antimicrobial drugs (unknown), antipyretics	Meropenem, azithromycin/2	Improved
11	45	Breast abscess	Fever for 10 d, right breast swelling	Diabetes mellitus	Incision and drainage/ pus aspirate	*Salmonella* Typhi/XDR strain	Antipyretics	Meropenem, azithromycin/2	Improved

*ID, identification; IM, intramuscular; MRSA, methicillin-resistant *Staphylococcus aureus*; NA, not available; XDR, extensively drug resistant..

## Conclusions

Our study was a comparison of isolation rates of XDR and non-XDR *Salmonella* Typhi from extraintestinal organ infections after the recognition of an XDR *Salmonella* Typhi outbreak in Pakistan. Our laboratory data highlights that, although isolation of XDR *Salmonella* Typhi from blood cultures has been performed since 2016, emergence and detection of XDR *Salmonella* Typhi from other body sites started during 2018, indicating a lag period of 14 months between the outbreak and extraintestinal organ infections. Except for 1 case of postileal perforation and collection of intraabdominal material, most of these manifestations were probably secondary to bacteremic seeding at various body sites. According to disease pathogenesis, different factors can contribute toward typhoid complication, including host-related factors, such as defects in innate immunity, any existing scar in soft tissue, inappropriate use of antimicrobial drugs, and organism- related virulence factors (*12*).

In this study, apparent immune dysfunction was found in only 2 patients: a 45-year-old woman who had a breast abscess and diabetes mellitus, and a 15-year-old boy who had splenic abscess and autoimmune hepatitis. A 55-year-old patient had a history of splenic injury and later showed development of a splenic abscess. Inappropriate antimicrobial drug use (Table 2) a common finding for most uncomplicated enteric fever cases, could be another contributing factor for uncontrolled disease process, leading to complications. However, this factor cannot solely be identified as a risk factor for these complicated cases. Another finding was that 4 of 11 extraintestinal organ infections were in adults, despite enteric fever being a disease with highest occurrence among children. We suggest future cohort studies to determine the reason of this delay in appearance and distribution of complicated cases.

Our data showed that during 2013–2018, irrespective of susceptibility pattern, the proportion of complicated enteric fever cases in Pakistan decreased (Table 1). Nonetheless, this finding could be a false impression and might be caused by an extraordinary increase in number of blood cultures requested during recent years. In Pakistan, laboratory diagnosis of enteric fever is commonly made on the basis of either positive serologic test results or blood culture. In view of low sensitivity and specificity of available serologic tests, our clinical laboratory purposely removed these isolates from its test procedure during 2015. Thus, blood culture is currently used as the main tool for diagnosis of enteric fever. During 2017, the number of XDR *Salmonella* Typhi cases increased from 21 during 2016 to 526 during 2017, but there was no similar emergence of XDR *Salmonella* Typhi in deep-seated infections.

Whole-genome sequencing of initial outbreak XDR isolates identified a plasmid encoding resistance elements, including the *bla*_CTX-M-15_ extended-spectrum β-lactamase carrying the *qnrS* fluoroquinolone resistance gene. This IncY plasmid exhibited high sequence identity to plasmids found in other enteric bacteria isolated from widely distributed geographic locations (*13*). However, in our study, molecular analysis was not performed, which is a limitation. In addition, our study was retrospective and single-laboratory based. Therefore, our study does not reflect the experience from other centers.

There was no increase in the proportion of XDR *Salmonella* Typhi extraintestinal isolates compared with non-XDR isolates. Because of the high endemicity of XDR *Salmonella* Typhi in Pakistan, general practitioners in outpatient clinics usually start empirical treatment for enteric fever on the basis of clinical judgment and serologic investigations. Enteric fever clinical practice guidelines specific to Pakistan are available (*14*) and should be followed to avoid complications of the disease. Lack of physician awareness regarding extraintestinal seeding of *Salmonella* Typhi can lead to inappropriate treatment. Thus, culturing of organisms is recommended before starting treatment.
